# Patient-Centered Self-Management in Patients with Chronic Kidney Disease: Challenges and Implications

**DOI:** 10.3390/ijerph17249443

**Published:** 2020-12-16

**Authors:** Chiu-Chu Lin, Shang-Jyh Hwang

**Affiliations:** 1 School of Nursing, Kaohsiung Medical University, Kaohsiung 80708, Taiwan; 2 Department of Renal Care, College of Medicine, Kaohsiung Medical University, Kaohsiung 80708, Taiwan; 3 Department of Medical Research, Kaohsiung Medical University Hospital, Kaohsiung Medical University, Kaohsiung 80708, Taiwan; 4 Division of Nephrology, Department of Internal Medicine, Kaohsiung Medical University Hospital, Kaohsiung Medical University, Kaohsiung 80708, Taiwan; sjhwang@kmu.edu.tw; 5 Faculty of Medicine, College of Medicine, Kaohsiung Medical University, Kaohsiung 80708, Taiwan; 6 Taiwan Society of Nephrology, Taipei 10022, Taiwan

**Keywords:** chronic kidney disease, empowerment, health-literacy, integrated care, patient-centered, self-management, self-regulation

## Abstract

This review aims to identify attributes of patient-centered self-management (PCSM) in the current literature and explore its implementation in resolving patient obstacles in chronic kidney disease (CKD) treatment and management. A search of relevant articles and literature on PCSM, integrated care, and challenges of CKD management was conducted. Vital attributes of PCSM and current self-management interventions employed to resolve patient obstacles in CKD management were identified from inclusion studies. Findings affirm that PCSM strategies have positive effects on CKD management, but a lack of quality primary study, and long-term evidence presents the need for further development. Future research should focus on the development of a standardized and universal integrated PCSM model and a uniform system of data collection in the clinical setting. The difficulty of CKD management lies in how it is a comorbid and progressive disease. A pure biomedical approach is inadequate. Our review recommends that an integrated PCSM approach with health literacy and information technology intervention, which unifies and integrates patient education, can address the difficulties that are contributing to unsuccessful treatment outcomes. An integrated PCSM model should be implemented systematically and methodologically into future CKD management and health policies.

## 1. Introduction

Chronic kidney disease (CKD) is a worldwide health problem. According to the 2017 Global Burden of Disease Study, CKD ranked 12th in the list of causes of deaths worldwide, affecting 13.4% of the global population [1]. Moreover, all-age prevalence of CKD has increased by 29.3% since 1990 [1,2]. Taiwan has the highest prevalence and incidence of CKD in the world [3]. The prevalence of CKD is increasing as people are living longer lives while the global population continues to rise. Longevity exists side by side with population aging and chronic elderly illnesses, which are major concerns for governments worldwide, as they pose hefty social and financial burdens to the individual, society, and health systems [4,5]. Symptoms of CKD are inapparent at the early stages of the disease [6], which usually allows for it to progress rapidly to end-stage renal disease (ESRD). Living with ESRD can be burdensome, particular when the demand for dialysis treatment increases, causing overwhelming financial and mental liabilities for individuals, their families, and health systems [7]. Lifestyle changes and early interventions are central to preventing disease or deferring progression of disease. Such is the case for CKD, which can abruptly deteriorate if the patient’s lifestyle changes do not follow a strict regimen. Thus, precautionary prevention becomes a health care priority. Treatment of CKD is focused on the prevention of disease progression towards ESRD, and one of the key factors of prevention is self-management (SM).

The concept of SM is central to many chronic conditions, as it is the gold standard of chronic care, and CKD is a classic example. Patients with chronic diseases usually have to live their entire lives with their disease. Thus, the treatment goal lies in improving their quality of life through SM. Chronic disease SM refers to an individual’s ability to handle the various aspects of living with a chronic condition [8,9], as well as “the everyday tasks an individual must undertake to control or reduce the impact of disease on physical health status [10,11].” Risk factors that predispose an individual to CKD can directly contribute to its severity and progression, but are modifiable through early detection and prevention through SM. These risk factors are usually lifestyle factors, primarily determined by the individual’s behavior and often shape the outcome of treatment. The impact of patients’ behavioral choices places patient-centered self-management (PCSM) at the core of chronic care and prevention models.

Patient-centered care (PCC) is a fundamental approach for improving the quality of health care and a promising paradigm where the patients’ values, goals, needs, and preferences are taken into consideration in the process of decision-making and delivery of health care [12]. A pure biomedical approach to chronic disease management does not adequately assess the health status of patients, and biomedical characteristics and biomarkers are often inadequate for predicting the patient’s actual wellbeing. Multiple facets of individuals and their environment must be considered in the treatment of disease, which denotes that a PC approach is vital in attaining effective SM in CKD treatment. Considering patient experience and standpoint can improve the accuracy of assessment and prediction in the treatment of disease. This gave rise to health frameworks with a focus on PCC (the PCC paradigm), which offers a much more comprehensive and all-rounded approach to understanding the patients’ overall wellbeing. In this review, the PCC paradigm is the framework that guides our review and findings. This review aims to explore attributes of PCSM in CKD in current literature, evaluate the common challenges, difficulties, and needs of implementing PCSM on CKD in research and clinical settings for both patients and health professionals (HPs), and offer recommendations and future directions for a more wholesome PCSM model that future studies may address.

## 2. Methods

This study aims to identify the attributes of PCSM in the current literature and explore its implementation in resolving patient obstacles in CKD treatment and management. This paper is a synthesis of literature rather than a systematic review. A search of relevant articles and literature on PCSM and challenges of CKD management was conducted using CINAHL, PubMed/MEDLINE, Cochrane, ProQuest, Ovid, and Google Scholar. Search terms include the following: *empowerment, health literacy, integrated care, patient-centered, self-management, self-regulation, chronic kidney disease, and patient-centered self-management*. Published qualitative and quantitative studies, abstracts or dissertations describing SM interventions in CKD patients, and published in English between 1985 and 2020, were included. Prominent attributes of PCSM (multidimensional concept, self-regulation, problem solving, and illness representation) and current SM interventions employed to resolve patient obstacles in CKD management were identified through synthesis of inclusion studies. Prominent attributes are defined as attributes that repeatedly and consistently appear or are mentioned in literature.

## 3. Results

In this section, we presented attributes of PCSM, highlighted their active elements, and identified current PCSM interventions. Then, we reported the implementation of CKD care from which existing challenges were identified, and proposed recommendations for future direction.

### 3.1. Definition of Patient-Centered Self-Management

The term self-management is often used broadly and is defined by a plethora of definitions and conceptualizations, while there is currently a lack of consensus on the definition of PCSM. So, we seek to establish conceptual clarity by identifying its most pronounced attributes through synthesis of the current literature. SM and PCC paradigm are defined separately below for clarity and to note its distinction.

#### 3.1.1. Definition of Self-Management

SM has been explored diversely in the existing literature. Lorig and Holman [13] (p. 11) defined SM as “learning and practicing skills necessary to carry on an active and emotionally satisfying life in the face of a chronic condition”. Barlow, Wright, Sheasby, Turner, & Hainsworth [8] (p. 178) depicted SM in-depth as “An individual’s ability to manage the symptoms, treatment, physical, psychosocial consequences, and lifestyle changes inherent in living with a chronic condition”. Lorig [14] further emphasized that “Self-management is not an alternative to medical care; however, it aims at assisting the individual in becoming an active, not adversarial, partner with HPs”. SM is generally regarded as where patients actively participate in day-to-day health-related activities, integrate therapeutic regimens into their daily lives, and live well with their illnesses. Overall, SM abilities can be defined as follows: participating in education/training to procure specific outcomes; preparing individuals to manage their health on a daily basis; practicing specific health behaviors; and having the skills and ability to deal with the physical and emotional impact of illnesses [15].

#### 3.1.2. Patient-Centered Care Paradigm

CKD is a progressive disease and often involves multiple co-morbidities, like hypertension, diabetes, and/or hyperlipidemia, making CKD SM difficult. Thus, SM programs for patients with CKD require an emphasis on PCC, which involves active participation and a positive change of patients’ attitude [16], while HPs offer personalized patient education support aimed at improving patients’ self-regulation abilities [6,17]. SM differs from the traditional paradigm in which patient compliance or adherence is the focus. The traditional notion of compliance with HPs’ orders resulted in a lack of adherence on the patient’s part to behavioral or lifestyle modifications during treatment [18]. The ultimate purpose of PCSM is to empower patients to manage their own disease and to navigate the health care system effectively [19]. SM urges patient autonomy rather than have them comply with and adhere to HPs’ orders [18,20]. In SM, patients play an active role in making decisions on their treatment and health management. Patient’s role shifts from being a passive compliant follower to becoming a proactive self-manager of his or her illness [20]. A PCSM model differs from the traditional HP-centered compliance paradigm, in which the person as a whole is regarded instead of merely treating the disease. Treating the patient as a person and not just their illness is an essential facet of this approach.

### 3.2. Attributes of Patient-Centered Self-Management

Pronounced attributes of PCSM were identified from inclusion studies, which consist of multidimensional concept, self-regulation, problem solving, and illness representation, as described below.

#### 3.2.1. Multidimensional Concept

Barlow et al. [8] reviewed prior studies and indicated that SM is a multidimensional concept combining “biological, psychological and social interventions techniques, with a goal of maximal functioning of regulatory process” [21]. The scope of SM is broad, covering multiple dimensions of a patient’s life, and is not merely confined to the medical realm. Corbin & Strauss [22] specifically addressed the intricate scope of SM by classing three dimensions of the SM process—medical, role, and emotional management [13,15,22,23]. To balance the three dimensions, patients acquire health-related knowledge, procure SM behaviors and abilities, and integrate recommended regimens into daily routines [9,24] through the process of self-regulation [25].

#### 3.2.2. Self-Regulation

Self-regulation is a key element of SM [17], in which patients self-monitor their behaviors, manage tasks, and use recorded information to achieve desired goals [25]. Individuals learn the skills required to handle a disease and its risk factors through the process of self-regulation [10]. Based on Bandura [26], self-regulation includes three processes that influence one another: self-monitoring, self-judgment, and self-reaction. Patients with chronic illnesses should learn to self-monitor their health issues through observing and understanding the consequences of their behaviors [26]; self-judge whether the current plan is effective; and self-react by either continuing with the current plan of action or reworking a new strategy [17]. In brief, self-regulation is a process of dynamic and reciprocal interaction. It refers to the ability to observe one’s own behavior *(self-monitor*), evaluate its effect (*self-judge*), and make amendments (*self-react*)” [27]. SM is an active, flexible, and continuous problem-solving process, in which patients develop strategies for achieving desired goals through utilizing self-regulation, collaborate with health care providers and significant others, and implement preventive and therapeutic health-related activities [28]. The ultimate goal of SM is to balance illness control and daily-life routine. Those who can integrate the two tend to achieve better quality of life [29]. Self-regulation abilities can help patients achieve integration between life and illness to deal with the conflicts of daily life and illness control.

#### 3.2.3. Problem Solving

Patients with chronic illnesses manage their disease on a daily basis amidst other personal concerns, such as life goals, priorities, health issues, and family demands [30]. Besides technical skills, problem-solving skills are required in disease management to manage daily barriers, adhere to recommended regimens, and make appropriate adjustments [31]. Patients learn to identify symptoms and apply SM strategies to manage their symptoms, through process of problem-solving. Problem solving skills should be integrated into clinical practice via a systematic and structured manner. Curtain et al. [32] proposed two domains in SM. The first domain is health care management, where HPs aid patients in acquiring “problem-solving ability,” in which patients are guided to identify problems and take initiative in learning pertinent knowledge and skills through patient empowerment. The methods are then refined and modified according to the results of their behaviors. The second domain focuses on implementing these learned abilities and illness prevention plan into the patient’s everyday life. The goal is to attain a balance between coping with illness and maintaining quality of life.

#### 3.2.4. Illness Representation

Illness representation, which is a patient’s beliefs and expectations of an illness, is correlated with how patients manage their illnesses [33]. Empirical studies have indicated that illness representation of patients with CKD affect their illness perceptions and coping behaviors [33,34]. Specifically, Leventhal et al. [34] proposed that individuals’ illness perception can influence their response and coping strategies when dealing with threats of illness. SM is a complex concept that requires an in-depth understanding of individuals’ sociocultural backgrounds in order to provide practical and individualized support. Sociocultural backgrounds can affect the patient’s decision-making on illness management [33]. Studies suggest that SM intervention can effectively slow disease progression in patients with CKD [35,36]. However, the patients’ willingness to adopt SM behaviors largely depend on how they perceive their disease or treatment [37,38]. Based on studies by the first author of this paper [37,38,39], patients with CKD often feel stigmatized due to cultural misconception, prompting them to conceal their illnesses, avoid or reject recommended treatment and pursue a variety of complementary therapies. Misconceptions of kidney disease and its treatments have made the coping process particularly difficult for patients. Thus, implementing a comprehensive assessment of a patient’s illness representation, usually shaped by factors like cultural diversity and sociocultural background, into standardized treatment is recommended. Knowing the patient as a person allows HPs to understand what is crucial to the patient’s adherence, so the PCC approach states the importance of allowing patients to have the opportunity to share their illness experiences [10,26].

### 3.3. Current Self-Management Interventions on CKD in Literature

We identified the attributes of PCSM in the previous section and will explore its interventions on CKD treatment and management in current literature in this section. Current SM interventions on CKD mainly help patients learn to identify symptoms, develop strategies to manage their symptoms, and implement good communication with HPs [40,41]. HPs are also recommended to adjust patient care from traditional paradigms to SM techniques [40,42] that emphasize a patient-centered (PC) approach [43,44,45].

Anderson’s empowerment model [46] is an approach to PC collaborative care where HPs work with patients to help them develop self-awareness of their concerns or needs; find barriers that impede lifestyle changes; learn the knowledge and skills needed to manage their diseases; and utilize resources to solve problems [47]. This empowerment model involves facilitating and supporting patients to reflect on their experiences, in which HPs help patients discover and develop their inherent capacity to gain mastery over their disease [48]. They learn to think critically and make informed decisions. As patients are in control of their daily SM decisions, they learn to be responsible for these decisions and the resulting consequences [49]. This approach does not involve convincing, persuading, or changing patients. Instead, it encourages doing things “with” patients and not doing things “for” them. Anderson’s empowerment model well connects the concept of PCC.

Havas et al. [43] stated that the principle of a PC approach is to improve the SM abilities of patients with CKD. In terms of patient perspective, Havas et al. [33] identified ten important themes that patients found were important in SM, including managing medications, engaging and sustaining social support, maintaining social and occupational roles, modifying lifestyle, developing and sustaining a positive attitude, and caring for mental and physical wellbeing, building and sustaining effective relationships with HPs, establishing routine and planning ahead, actively participating in healthcare, recognizing and effectively responding to systems, and acquiring disease-specific knowledge. These themes are important references for the future development of SM programs. Havas et al.’s study also showed that daily strategies should be prioritized in patient education. Different levels of engagement and eagerness to learn more of SM highlight the need for a PC approach to SM support.

To assess SM behaviors of early stage CKD patients, Lin, et al. [50] developed a CKD-SM instrument with multiple dimensions, which includes self-integration, problem-solving, seeking social support, and adherence to recommended regimen. Psychometric evaluation indicated that this instrument is a valid and reliable tool that has been authorized by the first author of this paper to the American Psychological Association and widely used by researchers. Investigators can use the CKD-SM instrument to identify areas of difficulty when patients self-manage their illness and further assist HPs in developing PC interventions to meet their diverse needs [50].

### 3.4. Challenges of CKD Management

We reviewed the literature on PCSM and challenges of CKD management, published between 1985 and 2020, to understand obstacles faced during the implementation and design of SM intervention programs in the treatment of CKD, and identified the following challenges in existing literature.

#### 3.4.1. Mental and Emotional Issues

Patients with chronic illnesses commonly suffer from emotional distresses, such as anxiety, insecurity, fear, and depression [51]. Literature we have reviewed regarding SM interventions on the mental challenges of CKD patients suggests that emotional management is a vital task for all patients with chronic diseases [51,52]. Lee, Wu, Hsieh, and Tsai’s [53] meta-analysis found that SM interventions can improve depression and patient’s mental quality, and that patients with CKD sought mental support in the management of CKD. Lin et al. [54] conducted a meta-analysis on the effects of SM on CKD and found medium effects on self-efficacy, depression, and health-related quality of life and a large effect on anxiety. This shows that SM can affect patient mentality and has potential in providing mental support and improving CKD management.

Likewise, Havas et al.’s [44] study on patients’ needs for SM support showed that patients display an avid interest in procuring aid in “keeping a positive attitude and taking care of mental and physical health” (p.3), validating that patients with CKD have a high demand for mental health support [53]. Overall, empirical studies in current literature have shown that SM interventions have a direct effect on patient mentality, especially in reducing anxiety. Thus, offering mental support and promoting close collaboration amongst patients and HPs is essential in reducing patients’ emotional distress and maintaining a healthy mentality [55].

#### 3.4.2. Lack of Organization and Unity in the Healthcare System

Havas et al. [44] found that patients with CKD experience a healthcare environment that is characterized by complexity and inconsistency. The reason is that the provision of SM support focused more on biomedical markers instead of on goals that are personally meaningful to patients. There is a lack of an organized and unified treatment scheme, resulting in conflicting advice from multiple HPs. A common issue, for example, is when patients must incorporate numerous healthcare appointments into their daily schedules without them overlapping and without interfering other health co-morbidities.

According to Green et al.’s [56] longitudinal research on the experiences of CKD patients in kidney disease management, patients felt that grasp of medical expertise, adherence to diet and fluid restrictions, management of medication, blood pressure control, interpretation of lab results, and decision-making on dialysis treatment were most challenging in terms of medical management.

In terms of patient’s grasp of medical expertise, patients tend to utilize diverse resources from health professionals, family, friends, educational courses, or the Internet. Ease of accessibility and home sessions enhanced patient’s active participation. Patients favored a single collective handbook of all CKD related materials to minimize chances of it getting lost and maximize the odds of it being used. While ease of accessibility and home sessions enhanced patient’s active participation. SM programs that were longitudinal, delivered in plain language, offered via multiple modalities (in-person, online, print), and with the help of peer support were preferred [56]. Studies showed that patients with CKD tend to utilize a variety of resources to assist them in managing their kidney disease [56]. Green et al. and Miller et al.’s studies revealed the need for long-term SM programs [7].

#### 3.4.3. Lack of Shared Decision-Making between HPs and Patients

According to Ong et al.’s [57] study, HPs and patients held contradictory views in terms of patient engagement in SM. While patients identified gaps in knowledge as a major obstacle, HPs believed patients lacked capacity and motivation. This misconception due to lack of proper communication resulted in inapt strategies that does not solve patient problems. Daker-White et al.’s [58] study showed that HPs-patient communication was more concerned with medical prescriptions in the primary care setting, where minimal opportunities were available for shared decision-making. This corresponded with Green et al. [56] that decision-making is a challenge in CKD management.

## 4. Discussion

### 4.1. Recommendations for Future Direction

We described important attributes of PCSM and its implementation on obstacles in CKD treatment and concluded that achieving success in treatment of CKD requires an integrated PCSM approach along with health literacy and information technology (IT) intervention, which unifies and integrates patient education. Synthesis of inclusion studies indicate that a pure biomedical approach to chronic disease management is inadequate, and that PCC is vital in attaining effective management. A focus on integrated PCC, health literacy, and IT can address the difficulties in SM interventions that may improve CKD treatment outcomes. A sequenced summary of integrated PCC, health literacy, and IT is as follows:

#### 4.1.1. Health Literacy

Patient’s knowledge and awareness of SM skills determine the effectiveness of treatment and is important in impeding advancement of CKD [54]. For patients to be effective at SM, they must possess the ability to utilize health information, a skill referred to as health literacy. Campbell et al. [59] indicated that low health literacy is associated with an increase in mortality and poorer overall health status of patients with CKD. While Grubbs et al. [60] stated that limited health literacy is associated with decreased access to transplantation, poor blood pressure control, and poor SM skills. Health literacy can impact a patient’s awareness and understanding of the importance of active participation in management of their illness. Put simply, health literacy can play a significant role in reducing the complications of CKD and preventing its progression [61].

Improving health literacy is the foundation for successful SM intervention [62], and health literacy can be developed through patient education [59]. Educational or therapeutic interventions used to promote patients’ level of health literacy are not only intended to reduce complications, but also to promote SM behaviors [59], and further improve patients’ quality of life [63]. Young [64] advises nephrologists to improve on their knowledge of the adverse effects of health literacy on patient health, and Campbell et al. [59] suggests fostering health literacy in patient education and implementing it into diverse SM interventions [54]. In an era of information explosion today, patients tend to utilize diverse resources from health professionals, family or friends, educational courses, or the Internet. Educating, detecting, and critically appraising health information of patients with CKD are some of the challenges faced in health literacy [65].

#### 4.1.2. Information Technology

IT is widely adopted in SM interventions nowadays to aid patients with CKD [66]. IT refers to mobile applications (apps), personal digital assistants (PDAs), wearable devices, computer systems, etc. IT is constructive in promoting education, empowerment, collaboration, and communication in SM [23,67], and offers HPs and patients a convenient and easily accessible platform where knowledge can be shared, and ideas can be exchanged. Specifically, apps helped patients in understanding specific biomarkers and self-monitoring of disease progression [68]. Web-based SM applications can be used to educate patients about CKD, monitor their progress, engage them to set goals, and provide an interactive tool for collaborating with clinicians in general [69,70]. IT interventions that target health behaviors, personal feedback mechanism, and collaborative platform between patients and HPs can lead to improved patient empowerment [61,70].

However, challenges were also identified in Ong et al. [69]’s study with a web-based SM application, called My Kidney Care Kiosk. Patients felt that the Kiosk application improved healthcare quality and strengthened interaction with HPs, but there were areas that needed improvements, such as a lack of integration of the application with the hospital electronic medical record system and high dependency on healthcare staff to promote the use of app. The change in staff workflow created increased workload and increased patient waiting time. Choi and Lee [6] cautions that direct, in-person interactions with HPs should not be replaced entirely by IT. Individualized consultations should be employed collectively with IT to address patient’s conditional needs.

#### 4.1.3. Integrated Patient-Centered Care

Integrated care is termed “a coherent set of methods and models designed to create connectivity, alignment, and collaboration within and between patients and health systems” [71]. Integrated care seeks to systematize and standardize a uniform and well-coordinated care system, in contrast to sporadic care, which contributes to unsuccessful treatment outcomes [72]. The goal of these methods and models is to enhance efficiency and reduce gaps in treatment of illnesses. It is the process by which professionals, resources, health sectors come together to form a comprehensive delivery of quality health care [71].

Patients with CKD often experience co-morbidity accompanying multiple interrelated illnesses (such as diabetes, cardiovascular disease, hypertension, obesity [52]). When these diseases are treated individually, it often leads to confusion and misperceptions, which builds on unnecessary costs and produces additional waste of medical resources. There is a need for further primary research on the effect of PC integrated care on patients with CKD that follows a uniform standard of data collection. The challenge is to develop a uniform and universal PC integrated care model that incorporates the interrelated co-morbidity factors that underlie the burden of CKD [72,73,74].

The lack of an organized and uniform method of data collection specifically focused on PC integrated CKD care management resulted in a marked lack of high-quality evidence available for literature reviews [74]. There was a lack of data available for organization-level care integration, methodologic limitations that reduced the confidence of data, a lack of long-term studies with follow-up beyond 12 months, and a lack of significant statistical values due to small sample sizes and low-quality data [72]. Future research direction should focus on establishing conceptual clarity and consolidate practical know-how with long-term follow-ups in the design and implementation of integrated care models.

### 4.2. Implications

**Figure 1:**
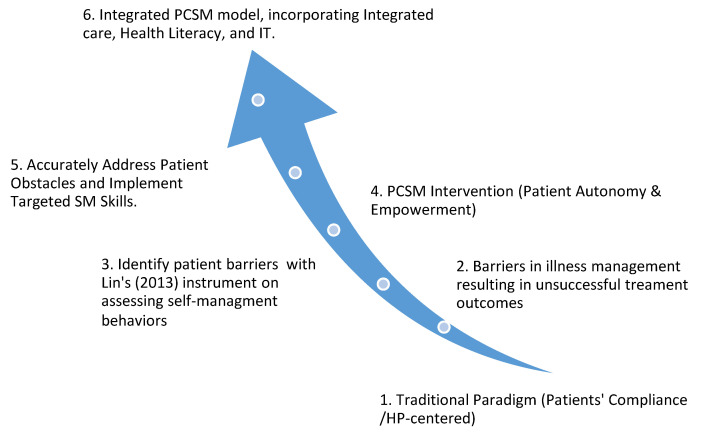
Progression from a traditional patient compliance paradigm to a more wholesome integrated patient-centered self-management (PCSM) model.

The difficulty of CKD management lies in that it is a comorbid and progressive disease, which can abruptly worsen without adequate lifestyle modification, early intervention, and long-term adherence. Thus, SM programs for patients with CKD require an emphasis on PC integrated care, which requires active participation and a positive change in patients’ attitudes [18], while HPs offer personalized patient education support aimed at improving patients’ self-regulation abilities [6]. Altogether, this review proposes that an integrated PCSM model with health literacy and IT intervention, which unifies and integrates patient education, can address the difficulties that are contributing to unsuccessful patient treatment outcomes. Achieving success in treatment of patients with CKD requires a shift from compliance paradigm to a PCSM approach, which comprises concepts like empowerment and self-regulation, along with health policies that incorporate systematic integrated care, while health literacy and IT intervention consolidate patient education. Figure 1 summarizes the main idea of this literature review and clarifies the process of evolving a more effective and wholesome integrated PCSM paradigm from the traditional paradigm, where patient compliance is the main focus.

## 5. Conclusions

We described important attributes of PCSM and its implementation in the obstacles of CKD patients during treatments and concluded that achieving successful long-term treatment requires an integration of SM skills with a focus on PC integrated care. All together, we believe that an integrated PCSM approach with health literacy and IT intervention, which unifies and integrates patient education, can address the difficulties that are contributing to unsuccessful patient treatment outcomes. The ultimate purpose of integrated PCSM is to empower patients in order to well-manage their own disease and to navigate the health care system effectively [16]. Studies have recommended HPs to shift patient care from traditional paradigms to SM techniques and emphasize a PC approach [43,44,45]. Current findings affirm that the PCSM approach can help resolve patient problems in disease handling through behavioral changes, but there is a lack of quality primary study and available evidence on the long-term effect of PCSM interventions in patients with CKD. Our review concludes that there is need for a standardized, universal, and wholesome integrated PCSM model and a uniform system of data collection in the clinical setting. It is recommended that an integrated PCSM model, along with health literacy and IT intervention, be implemented systematically and methodologically into future CKD management and health policies. Future research should focus on the development of a unified, valid, and reliable integrated PCSM model, with the aim of accurately addressing patient problems and subsequently implementing targeted SM skills.
